# Caffeic Acid Phenethyl Ester (CAPE) Induces VEGF Expression and Production in Rat Odontoblastic Cells

**DOI:** 10.1155/2019/5390720

**Published:** 2019-12-20

**Authors:** Hitomi Kuramoto, Kouji Hirao, Hiromichi Yumoto, Yuki Hosokawa, Tadashi Nakanishi, Daisuke Takegawa, Ayako Washio, Chiaki Kitamura, Takashi Matsuo

**Affiliations:** ^1^Department of Conservative Dentistry, Institute of Biomedical Sciences, Tokushima University Graduate School, 3-18-15 Kuramoto-cho, Tokushima 770-8504, Japan; ^2^Department of Periodontology and Endodontology, Institute of Biomedical Sciences, Tokushima University Graduate School, 3-18-15 Kuramoto-cho, Tokushima 770-8504, Japan; ^3^Division of Endodontics and Restorative Dentistry, Department of Oral Functions, Kyushu Dental University, 2-6-1 Manazuru, Kokura-kita, Kitakyushu, Fukuoka 803-8580, Japan

## Abstract

Caffeic acid phenethyl ester (CAPE), the main component of propolis, has various biological activities including anti-inflammatory effect and wound healing promotion. Odontoblasts located in the outermost layer of dental pulp play crucial roles such as production of growth factors and formation of hard tissue termed reparative dentin in host defense against dental caries. In this study, we investigated the effects of CAPE on the upregulation of vascular endothelial growth factor (VEGF) and calcification activities of odontoblasts, leading to development of novel therapy for dental pulp inflammation caused by dental caries. CAPE significantly induced mRNA expression and production of VEGF in rat clonal odontoblast-like KN-3 cells cultured in normal medium or osteogenic induction medium. CAPE treatment enhanced nuclear factor-kappa B (NF-*κ*B) transcription factor activation, and furthermore, the specific inhibitor of NF-*κ*B significantly reduced VEGF production. The expression of VEGF receptor- (VEGFR-) 2, not VEGFR-1, was up regulated in KN-3 cells treated with CAPE. In addition, VEGF significantly increased mineralization activity in KN-3 cells. These findings suggest that CAPE might be useful as a novel biological material for the dental pulp conservative therapy.

## 1. Introduction

Generally, dental caries first occurs on the enamel, the hard outer layer of teeth, and it progresses to dentin, the deep part of the teeth. Dental pulp tissues surrounded by dentin play functional roles in host defense against dental caries-related pathogens. As the tooth defect by dental caries approaches the dental pulp, the patient feels pain with cold and/or hot stimuli and pulpitis becomes apparent. With further progression of dental caries, pulpitis becomes irreversible and severe, and dental pulp removal therapy, called pulpectomy, is finally performed [1]. However, nonvital teeth without dental pulp tissues after pulpectomy have worse prognosis than vital teeth [2]. Therefore, to maintain dental pulp tissues is very important for the longevity of tooth.

In the current pulp conservative therapy, calcium hydroxide is mainly applied. To form reactive mineralized tissues termed dentin bridge, it is necessary to temporarily change the outermost layer of dental pulp tissues into the necrotic layer [3]. Therefore, the development of a more ideal dental pulp protective agent that promotes the formation of physiological dentin is expected. In order to develop a novel dental pulp conservative therapy, it is important to elucidate the detailed pathogenesis of pulpitis. Odontoblasts located in the outermost layer of dental pulp tissues recognize caries-related pathogens and possess a specialized innate immune system to fight their pathogens invading into dentin. We focused on the functions of odontoblasts and attempted to find out products applied for saving dental pulp tissues. However, the isolation and primary culturing of odontoblasts from dental pulp tissues have difficulty because of their limited numbers and the property of their replicative senescence. A rat odontoblastic cell line (KN-3) was established by limiting dilution cloning from dental papilla cells of lower incisors in Wistar rats [4]. KN-3 cells have high alkaline phosphatase activity and express odontoblastic cell markers such as dentin sialophosphoprotein (DSPP), dentin matrix protein-1 (DMP-1), and runt-related transcription factor 2 (Runx2) [4, 5]. In addition, KN-3 cells have the ability to form mineralized nodules [4, 5].

In general, immune responses in living organisms can be divided into innate and adaptive immunities. In the innate immune response, which is a rapid and antigen-independent host defense system, pattern recognition receptors (PRRs) expressed in host cells initially sense pathogen-associated molecular patterns (PAMPs) produced from microbial pathogens. Nucleotide-binding oligomerization domain (NOD) is well known as one of the innate immune receptors that recognize pathogenic factors. NOD1 and NOD2 present in the cytoplasm recognize the bacterial wall components *γ*-D-diaminopimelic acid (iE-DAP) and muramyl dipeptide (MDP), respectively [6, 7]. We have previously reported that KN-3 cells predominantly express NOD1, which may play important roles in the initial response of dental pulp tissues and progression of pulpitis [8].

Previous studies reported that polyphenols, such as catechin and caffeic acid, have an anti-inflammatory effect [9, 10]. Regarding the biological properties of polyphenols, various studies have reported that epigallocatechin-3-gallate (EGCG), a green tea catechin, has antioxidant effect as well as anti-inflammatory property [11] and we have also reported that EGCG has anti-inflammatory activity in human dental pulp fibroblasts [12]. Caffeic acid, mainly contained in coffee beans and fruits [13], has anti-oxidative, anticancer, and anti-inflammatory properties and effects of immunomodulatory actions [10, 14–16]. Caffeic acid phenethyl ester (CAPE) is a physiologically active substance of propolis, a resinous, sticky, coloured material prepared by honeybees to maintain aseptic conditions within the beehive using beeswax and plant exudates from certain plant sources [17, 18]. CAPE has many physiological effects such as inhibitory effect of cancer cell proliferation, inductive effect of apoptosis, improvement effect of tooth socket healing and bone healing, and anti-inflammatory effect [19–22].

The vascular endothelial growth factor (VEGF) A, simply VEGF, involves in angiogenesis by contributing to the growth of blood vessels, increases vascular permeability in order to compensate and meet the tissue demands [23, 24], and is also expressed in the dental pulp tissues [25]. Recent interesting studies have reported that VEGF involves in the activation of dental pulp stem cells, the differentiation of odontoblasts, and can induce the formation of reparative dentin [26]. However, there are no reports on the ability of polyphenols to induce hard tissues in odontoblasts.

In this study, we investigated the effects of polyphenols on the upregulation of VEGF and calcification activities of odontoblasts for the development of novel dental pulp conservative treatment.

## 2. Materials and Methods

### 2.1. Cell Culture

A rat clonal dental pulp cell line (KN-3 cells) with odontoblastic properties was cultured in alpha modification of minimum essential medium (*α*-MEM) (Life Technology, Carlsbad, CA, USA) normal medium containing 10% fetal bovine serum (Sigma-Aldrich, St. Louis, MO, USA), 100 U/ml of penicillin, and 100 *μ*g/ml streptomycin (Life Technologies) at 37°C in a humidified atmosphere of 5% CO_2_.

To determine the upregulation of VEGF and the mineralization activity, after KN-3 cells were seeded at 3 × 10^3^ cells/well in 24-well plates and cultured for 24 hours, KN-3 cells were cultured in osteogenic induction medium containing 10 mM *β*-glycerophosphate (Tokyo Chemical Industry, Tokyo, Japan) and 50 *μ*g/ml ascorbic acid (Wako, Osaka, Japan) for further 3 weeks until subconfluent.

### 2.2. Reagents

Caffeic acid and CAPE were purchased from Tocris Bioscience (Bristol, UK). EGCG was purchased from Sigma-Aldrich. iE-DAP was purchased from InvivoGen (San Diego, CA, USA). Recombinant rat tumor necrosis factor-*α* (TNF-*α*) was obtained from Peprotech (Rocky Hill, NJ, USA). SN50 was obtained from Santa Cruz Biotechnology, CA, USA. SR11302 was purchased from R&D Systems (Minneapolis, MN, USA). Recombinant rat VEGF was obtained from Wako.

### 2.3. Reverse Transcription-Polymerase Chain Reaction (RT-PCR)

Total RNA from KN-3 cells was isolated with a NucleoSpin RNA kit (Macherey-Nagel, Düren, Germany), and 100 ng RNA was utilized for each RT-PCR. RT and PCR were performed in two steps as follows. cDNA synthesis was performed using PrimeScript RT Master Mix (TaKaRa, Shiga, Japan), and specific gene transcriptions were amplified using ReddyMix PCR Mix (ABgene, Surrey, UK). The designs of PCR primers and reaction conditions are shown in Table 1. PCR products were analyzed by agarose gel electrophoresis and ethidium bromide staining.

### 2.4. Sodium Dodecyl Sulfate-Polyacrylamide Gel Electrophoresis (SDS-PAGE) and Immunoblot Analysis

KN-3 cells were cultured in 6-well plates and collected in RIPA lysis buffer (Santa Cruz Biotechnology). The protein concentrations in lysates were quantified using a bicinchoninic acid protein assay kit (Sigma-Aldrich). An equal amount of protein was loaded onto a 5–15% SDS-PAGE gel (Bio-Rad Laboratories, Hercules, CA, USA), followed by electrotransfer to a polyvinylidene difluoride membrane. The membrane was first incubated with DMP-1 antibody (TaKaRa) or DSP antibody (Santa Cruz Biotechnology). After washing, the membrane was reacted with horseradish peroxidase-conjugated secondary antibody (Sigma-Aldrich). Protein bands were finally visualized on X-ray film using the ECL Prime Western Blotting Detection system (GE Healthcare, Buckinghamshire, UK).

### 2.5. Lactate Dehydrogenase (LDH) Cytotoxicity Assay

KN-3 cells seeded in 24-well plates were treated with caffeic acid, CAPE, or EGCG in the concentration of 0.1 to 10 *μ*g/ml for 24 hours. Cytotoxic effect of caffeic acid, CAPE, and EGCG on the viability of KN-3 cells was assessed by observing the cell morphology under the microscope and quantified the amount of LDH released into the culture supernatant using LDH Cytotoxicity Assay Kit (Cayman Chemical, Ann arbor, MI, USA). Treatment with 0.1% Triton X-100 (Wako) for 10 minutes was used as a positive control.

### 2.6. PCR Array

Total RNA isolated from KN-3 cells treated with CAPE for 6 hours using a NucleoSpin RNA kit was reverse transcribed into cDNA using RT^2^ First Strand Kit (QIAGEN, Venlo, Netherlands) and analyzed the expression profiles of genes involved in bone metabolism, growth factors, and differentiation using RT^2^ Profiler™ PCR Array Rat Osteogenesis (QIAGEN) as described in the manufacturer's instructions.

### 2.7. Real-Time RT-PCR

Total RNA (20 ng) isolated from KN-3 cells with NucleoSpin RNA kit as described above was utilized for each real-time RT-PCR. Reverse transcription and real-time PCR were performed in two steps, as follows. cDNA synthesis was performed using PrimeScript RT Master Mix (TaKaRa), and specific gene transcriptions were amplified using Fast SYBR Green Master Mix (Thermo Fisher Scientific, Waltham, MA, USA) and a StepOnePlus Real-Time PCR system (Thermo Fisher Scientific). A housekeeping gene, glyceraldehyde-3-phosphate dehydrogenase (GAPDH), was used for sample normalization. The designs of PCR primers are shown in Table 2. For each target gene, relative expression was determined after normalization using the ΔΔCt method. Results were expressed as fold-change values relative to unstimulated control samples.

### 2.8. Enzyme-Linked Immunosorbent Assay (ELISA)

The concentration of VEGF in cell culture supernatant of KN-3 cells was determined using ELISA kit (R&D Systems) in accordance with the manufacturer's instructions.

### 2.9. Green Fluorescent Protein (GFP) Reporter Assay

A GFP reporter construct with transcriptional response element for nuclear factor-kappa B (NF-*κ*B) or activator protein-1 (AP-1), Cignal Reporter Assay Kit (QIAGEN), was transiently transfected into KN-3 cells using Attractene Transfection Reagent (QIAGEN) in accordance with the manufacturer's instructions, incubated for 24 hours, and then subjected to stimulation with CAPE for 24–48 hours. TNF-*α*, a representative of the proinflammatory cytokines, was used for positive control. The expression level of GFP was measured using a fluorescence microplate reader (Infinite® 200 PRO, Tecan, Männedorf, Switzerland).

### 2.10. Detection of Mineralization Activity

KN-3 cells were cultured in osteogenic induction medium with VEGF (100 ng/ml), and the medium was replaced every other day. KN-3 cells were treated with 0.05% Triton X-100 after 10-, 13-, and 17-day cultures, and their alkaline phosphatase (ALP) activity was determined by measurement of the absorbance at 405 nm with a microplate reader (Bio-Rad) using LabAssay ALP (Wako). Furthermore, after the removal of medium, the cells were fixed with 5% formaldehyde solution (Wako) for 60 minutes and then stained with 1% Alizarin red S staining solution (Wako) for 5 minutes. Quantification of the area occupied by calcium deposits from Alizarin Red S staining was analyzed by the measurement of absorbance at 405 nm using a microplate reader after the elution with 5% formic acid (Wako).

### 2.11. Statistical Analysis

All statistical analysis was determined by using the unpaired Student's *t*-test. Differences were considered significant when the probability value was less than 5% (*p* < 0.05).

## 3. Results

### 3.1. Expression of Odontoblastic Cell Markers in KN-3 Cells

**Figure 1:**
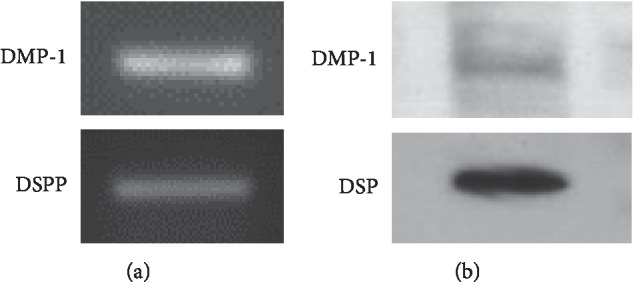
Expression of odontoblastic cell markers in KN-3 cells. (a) The gene expression of DMP-1 and DSPP in KN-3 cells was analyzed by RT-PCR. The results shown are representative images of two independent experiments with similar results. (b) The protein expression of DMP-1 and DSP in KN-3 cells was determined by immunoblotting analysis. The results shown are representative images of two independent experiments with similar results.

We first confirmed whether KN-3 cells express the odontoblastic cell markers using RT-PCR and immunoblotting analysis. The gene expression of DMP-1 and DSPP, encoding the protein of DSP, in KN-3 cells was detected (Figure 1(a)). The protein expression of DMP-1 and DSP in KN-3 cells was also verified (Figure 1(b)).

### 3.2. Cytotoxicity of Caffeic Acid, CAPE, and EGCG on KN-3 Cells

**Figure 2:**
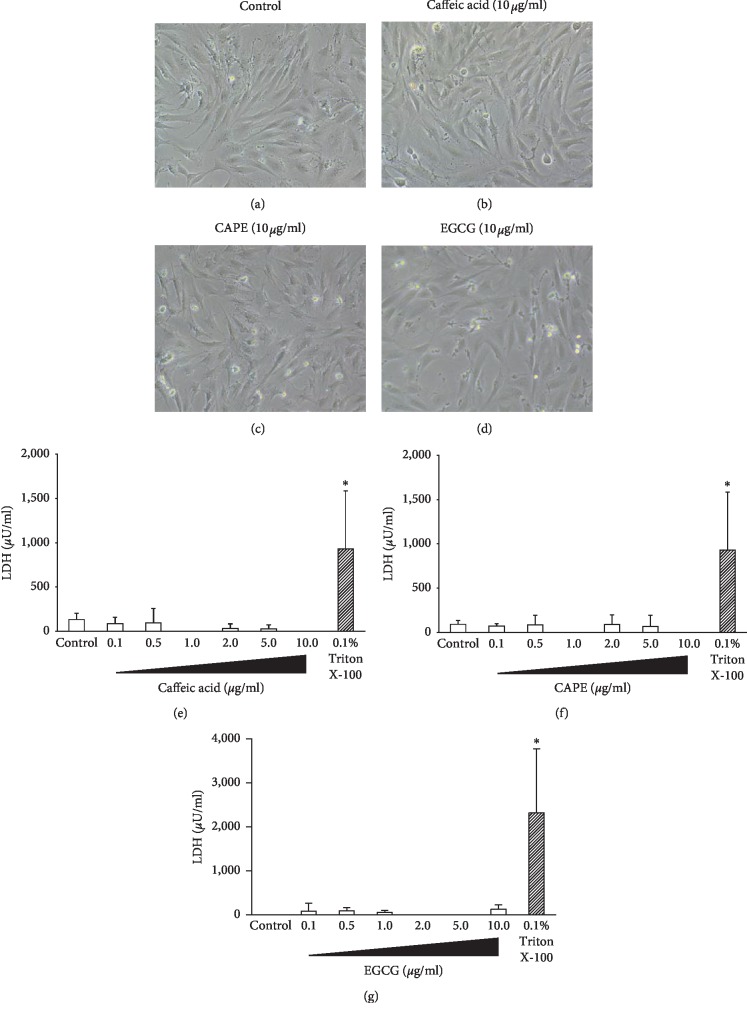
Cytotoxicity of caffeic acid, CAPE, and EGCG on KN-3 cells. KN-3 cells were treated with caffeic acid, CAPE, or EGCG (0.1, 0.5, 1.0, 2.0, 5.0, or 10.0 *μ*g/ml) for 24 hours. Microphotographs of KN-3 cells: nontreated control (without polyphenols) (a) and stimulated with 10 *μ*g/ml caffeic acid (b), 10 *μ*g/ml CAPE (c), or 10 *μ*g/ml EGCG (d) for 24 hours. Cytotoxicity of caffeic acid (e), CAPE (f), and EGCG (g) on KN-3 cells was analyzed by LDH cytotoxicity assay. Values represent the means ± SDs from representative of three independent experiments, and each experiment was performed in triplicate. Asterisks indicate significant differences versus the control without polyphenols (^*∗*^*p* < 0.05 vs. control).

The cytotoxicity of caffeic acid, CAPE, and EGCG on KN-3 cells was investigated by microscopic observation of cell morphology and LDH cytotoxicity assay. The specific morphologic change of KN-3 cells by caffeic acid, CAPE, and EGCG was not observed compared to the control (Figures 2(a)–2(d)). In addition, these polyphenols have no cytotoxic effect on KN-3 cell viability up to 10 *μ*g/ml (Figures 2(e)–2(g)).

### 3.3. Comprehensive Expression Analysis of Osteogenesis-Related Genes in CAPE-Treated KN-3 Cells

**Figure 3:**
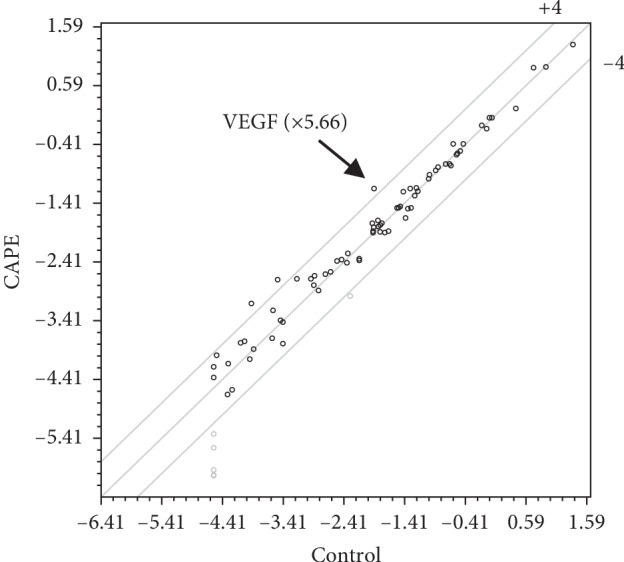
Comprehensive expression analysis of osteogenesis-related genes in CAPE-treated KN-3 cells by PCR array. Total RNA was isolated from KN-3 cells stimulated with CAPE for 6 hours in normal medium and reverse-transcribed into cDNA. The expression profiles of genes involved in bone metabolism, growth factors, and differentiation were analyzed using PCR array. The level of VEGF mRNA expression increased 5.66-fold by CAPE treatment.

In order to comprehensively analyze the expression of osteogenesis-related genes in CAPE-treated KN-3 cells, PCR arrays were performed. It was found that the mRNA expression level of VEGF increased 5.66-fold by CAPE treatment (Figure 3).

### 3.4. Expressions and Productions of VEGF in CAPE-Treated KN-3 Cells Cultured in Normal Medium and Osteogenic Induction Medium

**Figure 4:**
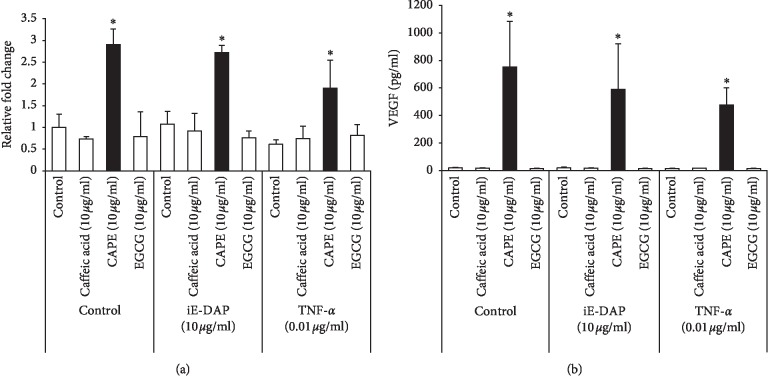
Expressions and productions of VEGF in CAPE-treated KN-3 cells in normal medium. KN-3 cells were stimulated with iE-DAP (10 *μ*g/ml) or TNF-*α* (0.01 *μ*g/ml) under the treatment with caffeic acid (10 *μ*g/ml), CAPE (10 *μ*g/ml), or EGCG (10 *μ*g/ml) for 6  and 24 hours in normal medium. (a) After 6-hour stimulation, total RNA was isolated and mRNA expression levels of VEGF were analyzed by real-time RT-PCR. Values represent the means ± SDs of three independent experiments, and each experiment was performed in triplicate. Asterisks indicate significant differences versus nontreated control (without polyphenols) (^*∗*^*p* < 0.05 vs. control). (b) The concentrations of VEGF in the cell culture supernatants after 24-hour stimulation were determined by ELISA. Values represent the means ± SDs of three independent experiments, and each experiment was performed in triplicate. Asterisks indicate significant differences versus nontreated control (without polyphenols) (^*∗*^*p* < 0.05 vs. control).

**Figure 5:**
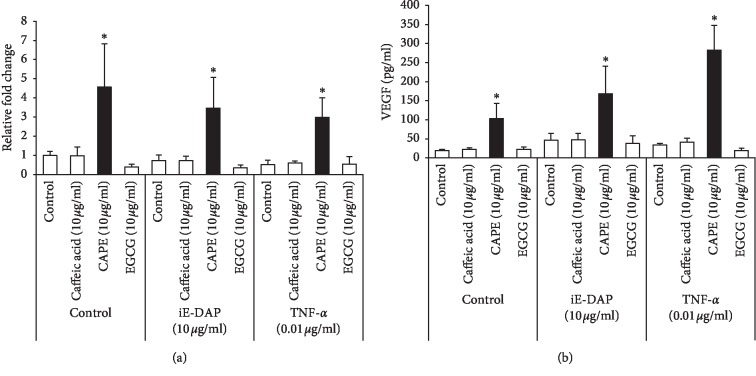
Expressions and productions of VEGF in CAPE-treated KN-3 cells in osteogenic induction medium. KN-3 cells were stimulated with iE-DAP (10 *μ*g/ml) or TNF-*α* (0.01 *μ*g/ml) under the treatment with caffeic acid (10 *μ*g/ml), CAPE (10 *μ*g/ml), or EGCG (10 *μ*g/ml) for 6 and 24 hours in osteogenic induction medium. (a) After 6-hour stimulation, total RNA was isolated and mRNA expression levels of VEGF were analyzed by real-time RT-PCR. Values represent the means ± SDs representative of three independent experiments, and each experiment was performed in triplicate. Asterisks indicate significant differences versus nontreated control (without polyphenols) (^*∗*^*p* < 0.05 vs. control). (b) The concentrations of VEGF in the cell culture supernatants after 24-hour stimulation were determined by ELISA. Values represent the means ± SDs representative of three independent experiments, and each experiment was performed in triplicate. Asterisks indicate significant differences versus nontreated control (without polyphenols) (^*∗*^*p* < 0.05 vs. control).

To confirm the inducing property of CAPE on both mRNA expression and protein production of VEGF in KN-3 cells, we performed real-time RT-PCR and ELISA, respectively, and compared the differences between CAPE and other polyphenols, such as caffeic acid and EGCG. Only CAPE was significantly able to induce both mRNA expression and production of VEGF in KN-3 cells cultured in normal medium (Figure 4). We also investigated whether iE-DAP or TNF-*α* could upregulate VEGF in KN-3 cells cultured with or without polyphenol. None of them had any effects on VEGF upregulation under all culture conditions. We next determined the effect of cell culture medium on CAPE-induced VEGF upregulation in KN-3 cells using osteogenic induction medium. CAPE significantly increased both mRNA expression and production levels of VEGF in KN-3 cells cultured in osteogenic induction medium similar to normal medium (Figure 5).

### 3.5. mRNA Expressions of VEGF Receptors in CAPE-Treated KN-3 Cells Cultured in Normal Medium and Osteogenic Induction Medium

**Figure 6:**
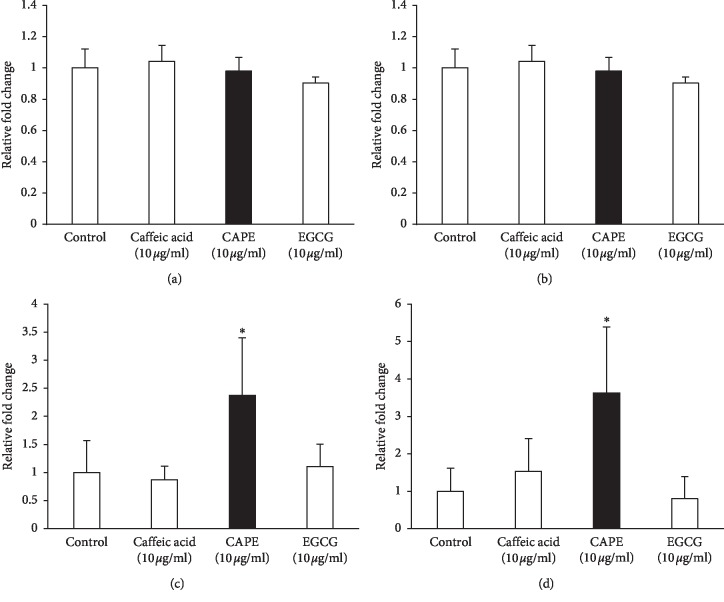
VEGF receptor mRNA expressions in CAPE-treated KN-3 cells in normal medium and osteogenic induction medium. KN-3 cells were stimulated with caffeic acid (10 *μ*g/ml), CAPE (10 *μ*g/ml), or EGCG (10 *μ*g/ml) for 6 hours in normal medium or osteogenic induction medium. After stimulation, total RNA was isolated and mRNA expression levels of VEGFR-1 and VEGFR-2 were analyzed by real-time RT-PCR. (a) VEGFR-1 mRNA expression levels in normal medium. (b) VEGFR-1 mRNA expression levels in osteogenic induction medium. (c) VEGFR-2 mRNA expression levels in normal medium. (d) VEGFR-2 mRNA expression levels in osteogenic induction medium. Values represent the means ± SDs representative of three independent experiments, and each experiment was performed in triplicate. Asterisks indicate significant differences versus the control (without polyphenols) (^*∗*^*p* < 0.05 vs. control).

We investigated the effect of CAPE treatment on the expression level of VEGF receptor in KN-3 cells under normal and osteogenic induction conditions using real-time RT-PCR. The level of VEGFR-1 mRNA expression was not induced by CAPE treatment under both culture conditions (Figures 6(a) and 6(b)). In contrast, CAPE treatment significantly induced VEGFR-2 mRNA expression under both culture conditions (Figures 6(c) and 6(d)). Caffeic acid and EGCG had no effects on the expressions of VEGFR-1 and VEGFR-2.

### 3.6. Cell Signaling Pathway Analysis in CAPE-Treated KN-3 Cells

**Figure 7:**
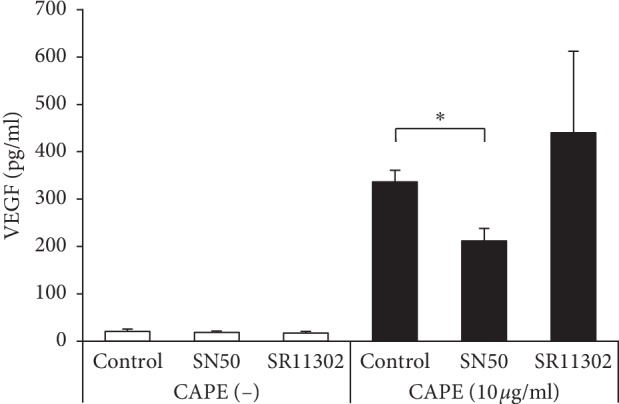
Cell signaling pathway analysis in CAPE-treated KN-3 cells. KN-3 cells were treated with SN50 (20 *μ*g/ml) or SR11302 (10 *μ*M) for 30 minutes followed by stimulation with CAPE (10 *μ*g/ml) for 24 hours. The concentrations of VEGF in cell culture supernatants were determined by ELISA. Values represent the means ± SDs representative of three independent experiments, and each experiment was performed in triplicate. Asterisks indicate significant differences versus the control (CAPE alone) (^*∗*^*p* < 0.05).

We investigated the cell signaling pathways involved in VEGF upregulation by CAPE-treated KN-3 cells using a specific inhibitor for signal pathway. The results from ELISA showed that CAPE-induced VEGF production was significantly suppressed by a NF-*κ*B specific inhibitor, SN50 (Figure 7). In contrast, the AP-1 specific inhibitor, SR11302, had no effect on the production of VEGF induced by CAPE treatment.

### 3.7. NF-*κ*B Activation in CAPE-Treated KN-3 Cells

**Figure 8:**
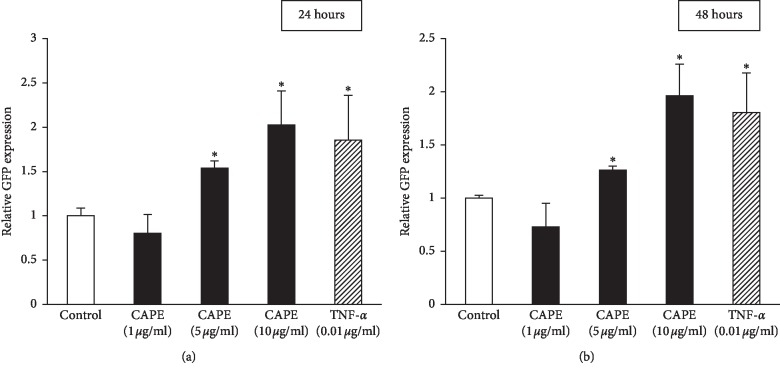
NF-*κ*B signal pathway in CAPE-stimulated KN-3 cells. A GFP reporter construct with transcriptional response element for NF-*κ*B was transiently transfected into KN-3 cells and subjected to stimulation with CAPE (1, 5,  or 10 *μ*g/ml) for 24 (a) or 48 hours (b). Treatment with TNF-α (0.01 μg/ml) was used as a positive control. The expression level of GFP was measured by using a fluorescence microplate reader. Values represent the means ± SDs from representative of two independent experiments, and each experiment was performed in quadruplicate. Asterisks indicate significant differences versus the control (medium alone) (^*∗*^*p* < 0.05 vs. control).

To confirm the role of NF-*κ*B in KN-3 cells treated with CAPE, GFP reporter assay was performed. GFP expression level controlled by NF-*κ*B transcriptional response was significantly upregulated by treatment with CAPE (5 *μ*g/ml and 10 *μ*g/ml) as well as TNF-*α* for the designated periods of time (24 and 48 hours) (Figure 8). We also examined the role of AP-1 in CAPE-treated KN-3 cells and found that GFP expression level controlled by AP-1 transcriptional response was not increased by neither CAPE or TNF-*α* treatment (data not shown).

### 3.8. Effect of VEGF on Mineralization Activity in KN-3 Cells under Osteogenic Induction Condition

**Figure 9:**
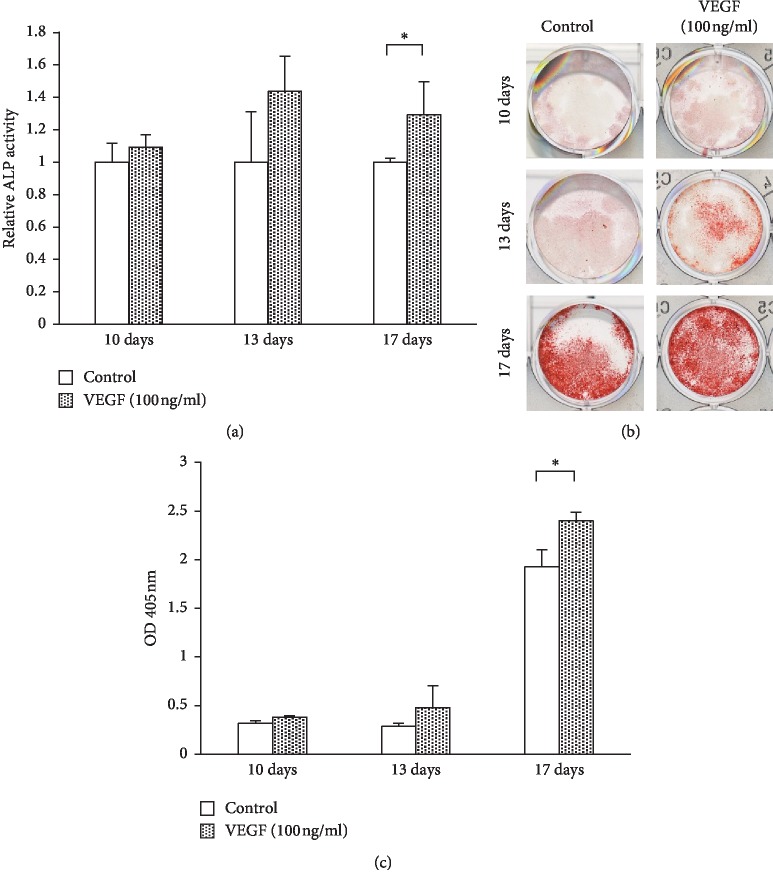
VEGF-induced mineralization activity in KN-3 cells under osteogenic induction condition. KN-3 cells cultured with osteogenic induction medium containing VEGF for 10, 13, or 17 days. (a) ALP activity was determined using LabAssay ALP (Wako) and normalized by protein amount. Values represent the means ± SDs from representative of three independent experiments, and each experiment was performed in triplicate. Asterisks indicate significant differences versus the control (without VEGF) (^*∗*^*p* < 0.05). (b) Alizarin Red S-staining of KN-3 cells cultured with VEGF (100 ng/ml) for 10, 13, or 17 days. (c) Quantification of the Alizarin Red S-stained area was analyzed by the measurement of absorbance at 405 nm using a microplate reader after the elution with 5% formic acid. Values represent the means ± SDs from representative of three independent experiments, and each experiment was performed in triplicate. Asterisks indicate significant differences versus the control (without VEGF) (^*∗*^*p* < 0.05).

ALP activity was significantly increased with VEGF treatment for 17 days under osteogenic induction condition, but not for 10 or 13 days in KN-3 cells (Figure 9(a)). Alizarin red staining also showed that mineralized nodule formation in the VEGF-treated KN-3 cells was significantly higher than that in the control group on day 17 (Figures 9(b) and 9(c)).

## 4. Discussion

Dental caries-related bacterial invasion induces destruction of dentin and inflammation of dental pulp. Vital pulp therapy is highly recommended for the treatment of deep dental caries close to pulpal exposure. The keys to success of vital pulp therapy are to eliminate infected bacteria, to generate good-quality of reparative dentin, and to control dental pulp inflammation. In this study, we focused on the roles of CAPE in activity of odontoblasts which participate in host defense such as reparative dentin formation in the dental pulp tissues.

The cytotoxicity of CAPE in the range of 10 *μ*g/ml concentration on odontoblast-like cells, KN-3 cells, was not observed. Consistent with the present observation, previous reports demonstrated that CAPE had no cytotoxicity for human umbilical vein endothelial cells (HUVEC) in almost the same concentration as our study [27, 28]. This finding suggests that the concentration of CAPE used in the present study could be clinically applied as a reasonable dose.

The dental pulp is a rich neovascular tissue [29]. Angiogenesis is an important step to efficiently recover the damaged dental pulp, and VEGF is the most potent angiogenic factor in repair of dentin [26]. This study for the first time demonstrated that CAPE treatment significantly induced upregulation of VEGF in KN-3 cells. Interestingly, the augmented VEGF production by CAPE was also observed even when proinflammatory factors, such as iE-DAP and TNF-*α*, were simultaneously exposed. A previous study reported that the expression of VEGF in the stromal cells in irreversible pulpitis was downregulated as compared with normal dental pulp [25]. This suggests that a local application of VEGF could be useful for the improvement of inflamed dental pulp. On the other hand, CAPE was demonstrated to have suppressive effect on VEGF-induced angiogenesis in HUVEC [30]. Hence, the control of VEGF production induced by CAPE treatment might be important in the clinical application to inflamed dental pulp tissues.

Among the receptors of VEGF (VEGFR-1, VEGFR-2, and VEGFR-3), VEGFR-2 mediates almost all VEGF-induced angiogenic effects, including microvascular permeability and angiogenesis [31, 32]. VEGFR-2 has been reported to be involved in many diseases such as cancer growth, metastasis through angiogenesis, and inflammatory diseases [33, 34]. In this study, the increased expression of VEGFR-2 as well as VEGF in CAPE-treated KN-3 cells was elucidated. VEGFR-2 is also known to be mainly expressed in the endothelial cells of dental pulp tissues [35]. In addition, a previous *in vitro* study suggests that the VEGF produced by dental pulp cells may promote cell differentiation into odontoblastic cells in an autocrine way via VEGFR-2 [36]. Taken together, the reinforcement of VEGF-VEGFR-2 action mediated by CAPE in an autocrine and/or paracrine manner might lead to hard tissue formation, such as dentin, and proliferation of blood vessels.

The anti-inflammatory effect of CAPE is suggested to be due to its selective inhibition of NF-*κ*B [37, 38]. Interestingly, our present study demonstrated that CAPE itself induced the activation of NF-*κ*B pathway leading to VEGF upregulation in KN-3 cells. This discrepancy may depend on the differences of cell type. In contrast, the AP-1 pathway was not related to VEGF production by CAPE, although we directed our attention to this signal pathway which was activated in KN-3 cells exposed to iE-DAP [8]. Transcription factors, such as NF-*κ*B and AP-1, play important roles in the regulation of cellular responses during inflammatory process. A previous study demonstrated that CAPE inhibited NF-*κ*B and AP-1 expressions induced by cytokine or *Helicobacter pylori* in gastric epithelial cells [39]. Our preliminary data showing that CAPE downregulated the production of proinflammatory cytokines in KN-3 cells stimulated with PAMPs also supported the anti-inflammatory properties of CAPE (data not shown). Therefore, the functions of CAPE on KN-3 cells might be influenced by the state of their cells.

Previous studies demonstrated that VEGF can potentiate ALP activity in human dental pulp cells and promote reparative dentin formation [26]. Consistent with their reports, ALP activity in KN-3 cells was significantly increased by VEGF in the present study. It has been shown that the higher activity of ALP, a marker for odontoblastic differentiation, was observed in the dental pulp cells at the early stage of calcification [40]. In our study, the upregulated ALP activity was found in the 17-day incubation with VEGF. This was in line with the result of Alizarin red staining showing that the mineralized nodule formation after 17-day exposure with VEGF was more prominent than that of the osteogenic medium alone. In addition, a drastic increase of the mineralized nodules at the incubation for 17 days as compared with for 13 days may indicate that the KN-3 cells enter into the activation stage for calcification in this period. These results suggest that VEGF could have capacity to induce KN-3 cells into more differentiated cells in respect of calcification.

## 5. Conclusion

In this study, we demonstrated that CAPE enhanced the production of VEGF in rat odontoblastic cells, KN-3 cells, through in part the signal transduction pathway for the activation of NF-*κ*B. Moreover, VEGF significantly enhanced mineralization activity in KN-3 cells. Our present findings in addition to previous findings regarding anti-inflammatory effects suggest that CAPE might be useful as a novel biological material for the conservative and regenerative treatment of inflamed dental pulp tissues.

## Figures and Tables

**Table 1:** Oligonucleotide sequences of primers used for RT-PCR.

Target gene	Primer	Sequence	Size of PCR fragment (bp)	PCR cycles	Annealing temperature (°C)
DMP-1	Sense	5′-GGACGGCTCTGAGTTCGA-3′	258	36	56
	Antisense	5′-TGGGTTTCCCTGCTGTTG-3′			

DSPP	Sense	5′-TCAATGGCGGGTGCTTTAGA-3′	110	40	60
	Antisense	5′-TGCTCACTGCACAACATGAAGA-3′			

DMP-1, dentin matrix protein- (DMP-) 1; DSPP, dentin sialophosphoprotein.

**Table 2:** Oligonucleotide sequences of primers used for quantitative real-time PCR.

Target gene	Primer	Sequence
VEGFA	Sense	5′-AAAAACGAAAGCGCAAGAAA-3′
	Antisense	5′-TTTCTCCGCTCTGAACAAGG-3′

VEGFR-1	Sense	5′-TACCTCACCGTGCAAGGAA-3′
	Antisense	5′-GAGTTAGAAGGAGCCAAAAGAGTG-3′

VEGFR-2	Sense	5′-CCCCAAATTCCATTATGACAA-3′
	Antisense	5′-GCTTTTTCGCTTGCTGTTCT-3′

GAPDH	Sense	5′-ACTCCCATTCTTCCACCTTTG-3′
	Antisense	5′-TGTAGCCATATTCATTGTCATACC-3′

VEGFA, vascular endothelial growth factor A; VEGFR-1, vascular endothelial growth factor receptor-1; VEGFR-2, vascular endothelial growth factor receptor-2; GAPDH, glyceraldehyde 3-phosphate dehydrogenase.

## Data Availability

The data used to support the findings of this study are available from the corresponding author upon reasonable request.
